# FEELING YOU BELONG: THE EFFECTS OF COMMUNITY INVOLVEMENT ON WELL-BEING IN LATER LIFE

**DOI:** 10.1093/geroni/igad104.2725

**Published:** 2023-12-21

**Authors:** Emily Briggs, Jacqui Smith

**Affiliations:** University of Michigan, Ann Arbor, Michigan, United States; University of Michigan, Ann Arbor, Michigan, United States

## Abstract

The area where someone lives is an important, yet neglected, contextual factor for the health of older adults. Previous literature suggests that an individual may develop an attachment to their neighborhood through a feeling of “rootedness,” which may contribute to feelings of belonging (Connerly & Marans, 1985). This sense of belonging is an essential component of harmonious communities with actively engaged residents. Together, feeling that you belong (perceived social cohesion) and active engagement in a community may be related to a higher overall sense of subjective well-being. Perceived social cohesion within a neighborhood also influences an individual’s level of community engagement. Using data from the Health and Retirement Study (HRS), this study investigates the impact of perceived social cohesion and community engagement on subjective well-being. The sample (N=5581, Mean Age (SD)= 68.34 (10.35), Range 50-98 years) included participants from the 2018 wave of data collection, who completed the Psychosocial Leave Behind Questionnaire. Multiple regression revealed that community engagement (β=.19, p=<.001) and social cohesion (β =.26, p=<.001) significantly predicted life satisfaction (R2=.096). These relationships remained significant after adjusting for age, gender, and years of education (Adj R2=.111, p<.001). These results suggest a sense of belonging may be more predictive than actual engagement on subjective well-being, this finding also interacts with age. Future work in this area should investigate the other possible influences that impact perceived feelings of belonging.

